# Insights from the Dalberto Teixeira Pombo (DTP) Arthropod Collection – I. Revealing the Hidden Diversity of Terrestrial Cave Arthropods in the Azores

**DOI:** 10.3897/BDJ.13.e158467

**Published:** 2025-09-10

**Authors:** Luís Carlos Fonseca Crespo, Fernando Pereira, Isabel R. Amorim, Paulo A. V. Borges

**Affiliations:** 1 University of the Azores, cE3c- Centre for Ecology, Evolution and Environmental Changes/Azorean Biodiversity Group, CHANGE – Global Change and Sustainability Institute, School of Agricultural and Environmental Sciences, Rua Capitão João d´Ávila, Pico da Urze, 9700-042, Angra do Heroísmo, Azores, Portugal University of the Azores, cE3c- Centre for Ecology, Evolution and Environmental Changes/Azorean Biodiversity Group, CHANGE – Global Change and Sustainability Institute, School of Agricultural and Environmental Sciences, Rua Capitão João d´Ávila, Pico da Urze, 9700-042 Angra do Heroísmo, Azores Portugal; 2 IUCN SSC Atlantic Islands Invertebrate Specialist Group, Angra do Heroísmo, Azores, Portugal IUCN SSC Atlantic Islands Invertebrate Specialist Group Angra do Heroísmo, Azores Portugal; 3 University of the Azores, CE3C/ABG – Centre for Ecology, Evolution and Environmental Changes & CHANGE - Global Change and Sustainability Institute / Azorean Biodiversity Group, Angra do Heroísmo, Portugal University of the Azores, CE3C/ABG – Centre for Ecology, Evolution and Environmental Changes & CHANGE - Global Change and Sustainability Institute / Azorean Biodiversity Group Angra do Heroísmo Portugal; 4 IUCN SSC Monitoring Specialist Group, Angra do Heroísmo, Azores, Portugal IUCN SSC Monitoring Specialist Group Angra do Heroísmo, Azores Portugal

**Keywords:** Arthropoda, island ecosystem, lava tube, Macaronesia, Troglobionts, volcanic pit

## Abstract

**Background:**

Hosted at the University of the Azores, the “Dalberto Teixeira Pombo” Collection (DTP) is an invaluable repository showcasing the diversity of arthropods from the Azores Archipelago, Portugal. This collection not only preserves a vital record of the region’s arthropological heritage but also underpins ongoing biodiversity research and conservation efforts. In this context, we are inaugurating a new series of Data Papers under the AZORES BIOTA Biodiversity Data Journal Collection. These papers will systematically document and analyze previously unidentified specimens derived from multiple past expeditions aimed at surveying and monitoring a range of habitats across the Azores Islands. By integrating historical collections with modern research methodologies, this initiative aspires to reveal previously hidden facets of the archipelago’s biodiversity and to inform future ecological, biogeographical and evolutionary studies, as well as conservation endeavours.

This first manuscript targets the subterranean arthropod fauna. Collected primarily during intensive field expeditions between 1991 and 2010, subterranean samples from the archipelago’s diverse cave systems and mesovoid habitats form a vast assemblage of specimens — most of which remain unidentified — that illuminate the region’s hidden and understudied biodiversity. Notably, the only taxonomically resolved subset comprises the cave-adapted ground‐beetles of the genus *Trechus*, a group of paramount importance to the understanding of the evolution of Azorean subterranean adapted fauna.

**New information:**

This study significantly contributes to addressing the Wallacean shortfall within the Azorean subterranean ecosystem by documenting novel range extensions for key endemic cave-adapted arthropods. Among the taxa recorded in previously uncharted locations are the centipede *Lithobius obscurus azoreae* Eason & Ashmole, 1992, the pseudoscorpion *Pseudoblothrus vulcanus* Mahnert, 1990, and several single-island endemic ground beetles of the genus *Trechus*, including *Trechus terceiranus* Machado, 1988 and *Trechus picoensis* Machado, 1988. These new distributional records enhance our understanding of species biogeography in the unique subterranean volcanic systems of the Azores and provide valuable insights into the connectivity and dispersal potential of troglobitic species in oceanic island settings.

Furthermore, this research plays a crucial role in defining new priority areas for the conservation of Azorean cave-adapted arthropods. By refining knowledge on species distributions, it supports the development of targeted spatial conservation strategies aimed at mitigating habitat degradation and preserving the fragile subterranean biodiversity of the archipelago. The findings of this study contribute directly to future conservation planning, ensuring that appropriate protective measures are implemented to safeguard these highly specialized and often vulnerable cave ecosystems.

## Introduction

Caves provide remarkable examples of a niche suitable for evolutionary phenomena, namely ecological, behavioural and physiological adaptation, operating at different scales (Culver and Pipan 2009). Their special abiotic conditions, such as reduced light, low thermal amplitude, high humidity, oligotrophic conditions and confined space often lead to the evolution of subterranean adapted species (troglobionts), which usually possess traits suitable for this kind of environment, such as low dispersal ability, low metabolic rate, loss of pigments and eyesight, and long appendages (Culver and Pipan 2009, Pipan and Culver 2012). An intermediate degree of adaptation to subterranean environments is given by troglophiles, organisms that can live their entire lives in subterranean habitats, but which also exist in other environments (Sket 2008). As a result of the low diversity of organisms occupying such an extreme environment, troglobionts often occupy key ecological roles in subterranean ecosystem (Parimuchová et al. 2021). The evolutionary process of the appearance of troglobionts is usually linked to the geological ontogeny of the subterranean habitats, and in the case of the cave system the two most known types are (Chapman 1993, Moseley 2009): a) *karst* formations, usually present in mainland masses, originating from erosional processes that gradually remove rock both at the surface and the underground, and b) lava tubes or volcanic pits, cavities originating from the passage of lava during the active volcanism (Howarth 1983).

**Figure 1.**
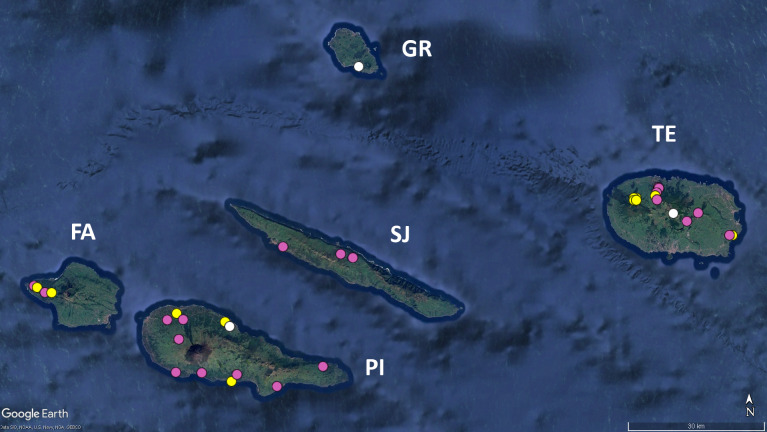
Map of the Central group of the Azores, with the distribution data of the endemic centipede *Lithobius obscurus azoreae* Eason & Ashmole, 1992. Legend: yellow circles: locations cited in literature; pink circles: locations recorded for the first time in this study; white circles - locations from unpublished sources. FA - Faial, GR - Graciosa, SJ - São Jorge, PI - Pico, TE - Terceira. Image retrieved from Google Earth.

The second type is often prevalent in islands with a volcanic origin, such as the Azores archipelago. The Azores are a group of nine islands situated in the central North Atlantic Ocean (37─40 °N, 25─31 °W) located roughly 1600 km from the Iberian Peninsula and 2200 from North America. The geological age of the archipelago dates from 6 Ma (Santa Maria) (Ramalho et al. 2016) to 190 ka (Pico) (Costa et al. 2015) (whole archipelago geological ages compiled in Florencio et al. 2021). The last available inventory lists a total of 272 volcanic cavities knwon from the Azores (Pereira et al. 2015), but a more recent, not yet published, inventory lists at least 350 cavities (P. Barcelos, personal comm.). The majority of Azorean caves are located in Pico and Terceira (Borges et al. 2012, 102: fig. 1), a few of them being also touristic attractions, such as Algar do Carvão and Gruta do Natal (Terceira) and Gruta das Torres (Pico). Borges et al. (2012) accounted up to 17 troglobionts in the archipelago, while attempting to score the conservation priority of the distinct caves of the Azores. Azorean caves are nowadays protected by law (overviewed in Mammola et al. 2024: 6, box 3) and are a prime example of positive outcomes derived from the joint effort of researchers, governmental stakeholders and regional ENGO's ("Os Montanheiros" and "Amigos dos Açores"). Moreover, each species profile was assessed from a conservationist standpoint by Borges et al. (2019), thus providing species specific baseline knowledge for conservation purposes (see e.g. Borges and Amorim 2018a, Borges and Amorim 2018b). These are necessary tools to have at hand when faced with several anthropogenic threats, such as direct damage from human activity. Two examples of this are the destruction of Gruta do Camelo (Terceira) due to accessing a spring’s aquifer, or Gruta do Soldão (Pico) due to industrial activity. Despite legal protection, damage continues to be inflicted on these fragile habitats. Moreover, climate change might negatively affect the otherwise stable conditions of these subterranean biomes, to which troglobionts are adapted (Mammola et al. 2019).

The knowledge on Azorean troglobionts has been biased by a few factors: first, accessibility. While some volcanic cavities are of somewhat easy access for a small crew of researchers (e.g. several lava tubes), others, such as deep volcanic pits, demand field trips composed of several experienced speleologists. Moreover, the survey of MSS (*Milieu Souterrain Superficiel* or Mesovoid Shallow Substratum) despite some successful results (Borges 1993) needs some more reserach investment in the Azores. As a result, regular biodiversity monitoring is only conducted in the easily accessible cavities. Second, taxonomic effort. Despite Azorean subterranean fauna being relatively well studied from the classical morphology-based taxonomic approach, molecular methods are still not integrated into taxonomic studies, except for the genus *Trechus* (Amorim 2005). One example of this is represented by the pseudoscorpion *Pseudoblothrus vulcanus* Mahnert, 1990, which presents a disjunct distribution with two populations, one in Terceira and the other on Pico; aditionally another species of the same genus is present in the close by island São Jorge, the single-island endemic (SIE) *Pseudoblothrus oromii* Mahnert, 1990 (Mahnert 1990, Borges et al. 2022). The status of the populations of *P. vulcanus* can only be revised via an integrative taxonomic study using molecular data (Zaragoza, personal comm.). Within the epigean realm, a recent integrative taxonomic revision of the beetle genus *Tarphius* exemplifies how combining morphological, molecular, and ecological analyses can lead to significant taxonomic refinements (Borges et al. 2017). In this study, Azorean endemics occurring in multiple islands were re‐evaluated and ultimately separated into several single island endemic species, thereby enhancing our understanding of species boundaries and evolutionary dynamics in insular ecosystems. Third, sampling standardization. The collection of subterranean biodiversity has been subject to distinct objectives and efforts across the past decades in the Azores. At first, mostly *ad-hoc* sampling was being conducted towards the novel taxonomic findings to be found, only later a concern towards standardization was implemented. As usual, a variety of protocols was used, depending on personal preferences, such as the number and types of traps, and the nature of the baits experimented on these (Oromí et al. 1990, Amorim 2005).

This manuscript reports on the biological materials of arthropods sampled by members and collaborators of the University of Azores since the 1990’s on subterranean environments of the Azores. We will highlight the occurrence of endemic species in new localities, therefore increasing their distribution range. These new records will update the conservation priority of the distinct subterranean environments, and records of unidentified taxa can call the attention of prospective taxonomists that might be keen on studying these materials.

## General description

### Purpose

The primary goal of this study is to present the findings from a comprehensive inventory of the arthropod subterranean fauna across the Azores Islands collected in the period between 1991-2010. In addition to documenting the distinctive species assemblages found in these subterranean habitats, the study also seeks to: i) urge policymakers, researchers, and conservationists to give priority to the protection of subterrranean ecosystems and their surrounding environments; and ii) emphasize the cultural and scientific significance of incorporating subterranean ecosystems into broader conservation strategies.

By meeting these objectives, the study aims to contribute meaningfully to biodiversity research, environmental education, and the sustainable management of these unique subterranean habitats.

### Additional information

The Azorean volcanic cavities and MSS provide an essential habitat for endemic troglobitic species that have evolved specialized adaptations to the underground environment, such as depigmentation, reduced eyes, and elongated appendages. Due to their isolation and the limited availability of suitable underground habitats, many subterranean-adapted species are highly vulnerable to habitat degradation. Conservation efforts in the region include classifying volcanic cavities, namely caves, into four priority levels (*Decreto Legislativo Regional n*.º 10/2019/A, *de 22 de maio* and *Resolução do Conselho do Governo n.º 163/2024 de 4 de novembro de 2024*).

## Project description

### Title

Inventory of arthropods in Azorean Islands subterranean environments: lava tubes, volcanic pits and MSS

### Personnel

The project was conceived and is being led by Isabel R. Amorim and Paulo A.V. Borges.

Fieldwork (site selection and experimental setting): Isabel R. Amorim, Fernando Pereira and Paulo A.V. Borges.

Fieldwork (authorisation): Azorean Regional Directorate for the Environment.

Parataxonomists (Laboratory): Fernando Pereira, Isabel R. Amorim and Luís Carlos Crespo.

Taxonomists: Luís Carlos Crespo and Paulo A.V. Borges.

Arthropod Curation: Luís Carlos Crespo.

Darwin Core Databases: Luís Carlos Crespo and Paulo A.V. Borges.

### Study area description

The Azores archipelago, located in the North Atlantic Ocean along the Mid-Atlantic Ridge (approximately 36°55′ to 39°43′ N and 24°33′ to 31°17′ W), consists of nine volcanic islands that host an extensive network of lava tubes and volcanic pits. The archipelago harbors approximately 350 lava tubes and volcanic pits, with Pico and Terceira islands containing the highest number, at 118 and 73 caves, respectively (Pereira et al. 2015). These caves vary in their geological composition, size, and environmental conditions, forming unique subterranean ecosystems that support specialized cave-adapted fauna, including endemic arthropods and microbial communities (Oromí et al. 1990, Borges et al. 2012, Borges et al. 2019). In this study we sampled 46 caves, listed below in Table 1.

### Funding

This research was funded by Biodiversa+ (project ‘DarCo’), the European Biodiversity Partnership under the 2021–2022 BiodivProtect joint call for research proposals, cofunded by the European Commission (GA N°101052342) and Fundo Regional para a Ciência e Tecnologia (Portugal). IAR was funded by national funds through FCT – Fundação para a Ciência e a Tecnologia, I.P., under the Norma Transitória https://doi.org/10.54499/DL57/2016/CP1375/CT0003.

Data availability for the general public is funded by AZORES BIOPORTA: PORBIOTA (ACORES-01-0145-FEDER-000072).

Materials stored in the "Dalberto Teixeira Pombo Collection were mostly collected under the scope of the Ph.D. work of Isabel R. Amorim financed by JNICT, UCLA and BALA project (Azorean Direcção Regional dos Recursos Florestais (Azorean Government / project 17.01-080203).

## Sampling methods

### Study extent

Volcanic cavities were selected based on their geological diversity, accessibility, and conservation status. Sampling efforts have included invertebrate surveys, targeting specially subterranean adapted arthropod species.

### Sampling description

The study of cave-dwelling fauna in the Azores utilized multiple standardized sampling methods to ensure comprehensive coverage of arthropod biodiversity across various cave systems. The primary methodologies included pitfall trapping and visual searching, each adapted to the unique environmental conditions of the Azorean lava tubes and volcanic pits.

We used pitfall traps as the most highly effective method for capturing cave-dwelling arthropods, particularly ground beetles of the genus *Trechus*. Following Amorim (2005):

- Trap Setup: Small plastic pitfall traps were placed along standardized transects inside lava tubes and volcanic pits. Each transect, whenever possible, consisted of 30 pitfall traps spaced along a 500-meter-long transect from the cave entrance inwards. Traps were dug into the ground and/or placed inside cracks on vertical walls, to maximize the chance of collecting arthropods with distinct lifestyles, and covered with mesh to protect against rats. To prevent flooding from dripping water, plastic plates were installed above traps.
- As Baiting Strategy several types of baiting were used: i) Pitfall (bait: liver) - live traps baited with fresh cow and/or pig liver that was kept in a special container covered with a mesh inside the traps, allowing the collection of live specimens; ii) Pitfall (bait: turquin) - live traps baited with TURQUIN (a mixture of 1 litter of dark beer and some preservatives: 5 ml Formaldehyde; 5 ml Glacial Acetic Acid; 10 g Sodium Chloride) (Turquin 1973) (see more details in Borges 1993) that was kept in a separate container covered with a mesh inside the traps, allowing the collection of live specimens; iii) Pitfall (bait: liver + cheese) - live traps baited with fresh cow and/or pig liver plus cheese that was kept in a separate container covered with a mesh inside the traps, allowing the collection of live specimens; iv) Pitfall (bait: turquin + cheese) - killing traps 1/3 filled with TURQUIN plus cheese that was kept in a separate container covered with a mesh inside the traps; v) Pitfall (bait: turquin + liver) - killing traps 1/3 filled with TURQUIN plus liver that was kept in a separate container covered with a mesh inside the traps. A piece of toillet paper was added to "live traps" to provide shelter to the live specimens that fell into the traps. Life specimens were preferable for DNA studies, but because some specimens were able to escape the "live traps" (e.g., spiders), "killing traps" were also set up to provide a better representation of arthropods that inhabit cave habitats.
- Pitfall traps were left in place for at least seven days and up to a month to ensure adequate specimen collection. Traps were monitored periodically to prevent desiccation or disturbance. Occasionally, longer trapping periods occurred due to climatic and logistical constraints.

Collected specimens were primarily stored in 100% acetone, known to be an optimal preservative for DNA analyses. For logistical reasons, some specimens were initially placed in 70% ethanol before identification and subsequently transferred to 100% acetone. Specimens were kept either at -20ºC or at room temperature. After species identification performed for this study all specimens were stored in 96% ethanol at room temperature.

### Quality control

Arthropod species taxonomic nomenclature follows Borges et al. (2022).

## Geographic coverage

### Description

This study covers seven of the nine Azorean islands (all but Corvo and Flores).

### Coordinates

36.862 and 39.13 Latitude; -28.872 and -24.741 Longitude.

## Taxonomic coverage

### Description

Phylum: Arthropoda

Class: Arachnida, Chilopoda, Diplopoda, Insecta

Order: Araneae, Chordeumatida, Coleoptera, Dermaptera, Diplura, Geophilomorpha, Hemiptera, Julida, Lithobiomorpha, Opiliones, Polydesmida, Pseudoscorpiones, Scutigeromorpha.

Family:

Anisolabididae, Aphididae, Blaniulidae, Campodeidae, Carabidae, Chthoniidae, Cicadellidae, Cixiidae, Cryptophagidae, Curculionidae, Dysderidae, Elateridae, Formicidae, Geophilidae, Haplobainosomatidae, Julidae, Leiobunidae, Leiodidae, Linyphiidae, Lithobiidae, Lygaeidae, Nitidulidae, Paradoxosomatidae, Pholcidae, Polydesmidae, Rhyparochromidae, Salticidae, Scutigeridae, Silvanidae, Staphylinidae, Syarinidae, Tenebrionidae, Tetragnathidae, Theridiidae.

Due to the lack of specialists on some Arthropod groups, a number of taxa still remain unidentified in the Dalberto Teixeira Pombo collection, namely, Amphipoda, Acari, Collembola, Diptera, Hymenoptera, Isopoda and Lepidoptera.

## Temporal coverage

**Data range:** 1991-6-07 – 2010-10-22.

## Collection data

### Collection name

Dalberto Teixeira Pombo /NCBI- BioCollections - UAC<PRT>:DTP

### Collection identifier

DTP

### Specimen preservation method

Ethanol96%

## Usage licence

### Usage licence

Creative Commons Public Domain Waiver (CC-Zero)

## Data resources

### Data package title

Inventory of arthropods in Azorean Islands lava tubes and volcanic pits

### Resource link

http://ipt.gbif.pt/ipt/resource?r=arthropods_azores_darco

### Alternative identifiers

https://www.gbif.org/dataset/4a3a1ea5-9ff6-459f-9b04-6ec577ef042e

### Number of data sets

2

### Data set 1.

#### Data set name

Event Table

#### Data format

Darwin Core Archive

#### Character set

UTF-8

#### Download URL

http://ipt.gbif.pt/ipt/resource?r=arthropods_azores_darco

#### Data format version

1.7

#### Description

The dataset was published in the Global Biodiversity Information Facility platform, GBIF (Borges et al. 2025). The following data table includes all the records for which a taxonomic identification of the species was possible. The dataset submitted to GBIF is structured as a sample event dataset that has been published as a Darwin Core Archive (DwCA), which is a standardised format for sharing biodiversity data as a set of one or more data tables. The core data file contains 600 records (eventID). This GBIF IPT (Integrated Publishing Toolkit, Version 2.5.6) archives the data and, thus, serves as the data repository. The data and resource metadata are available for download in the Portuguese GBIF Portal IPT (Borges et al. 2025).

**Data set 1.** 

Column label	Column description
eventID	An identifier for the set of information associated with a dwc:Event (something that occurs at a place and time). May be a global unique identifier or an identifier specific to the data set.
samplingProtocol	The names of, references to, or descriptions of the methods or protocols used during a dwc:Event.
sampleSizeValue	A numeric value for a measurement of the size (time duration, length, area, or volume) of a sample in a sampling dwc:Event.
sampleSizeUnit	The unit of measurement of the size (time duration, length, area, or volume) of a sample in a sampling dwc:Event.
samplingEffort	The amount of effort expended during a dwc:Event.
eventDate	The date-time or interval during which a dwc:Event occurred. For occurrences, this is the date-time when the dwc:Event was recorded. Not suitable for a time in a geological context.
year	The four-digit year in which the dwc:Event occurred, according to the Common Era Calendar.
month	The integer month in which the dwc:Event occurred.
day	The integer day of the month on which the dwc:Event occurred.
habitat	A category or description of the habitat in which the dwc:Event occurred.
fieldNumber	An identifier given to the event in the field. Often serves as a link between field notes and the dwc:Event.
islandGroup	The name of the island group in which the dcterms:Location occurs.
island	The name of the island on or near which the dcterms:Location occurs.
country	The name of the country or major administrative unit in which the dcterms:Location occurs.
countryCode	The standard code for the country in which the dcterms:Location occurs.
stateProvince	The name of the next smaller administrative region than country (state, province, canton, department, region, etc.) in which the dcterms:Location occurs.
municipality	The full, unabbreviated name of the next smaller administrative region than county (city, municipality, etc.) in which the dcterms:Location occurs. Do not use this term for a nearby named place that does not contain the actual dcterms:Location.
locality	The specific description of the place.
minimumElevationInMeters	The lower limit of the range of elevation (altitude, usually above sea level), in meters.
locationID	Identifier of the locations, unique for the dataset.
decimalLatitude	The geographic latitude (in decimal degrees, using the spatial reference system given in dwc:geodeticDatum) of the geographic center of a dcterms:Location. Positive values are north of the Equator, negative values are south of it. Legal values lie between -90 and 90, inclusive.
decimalLongitude	The geographic longitude (in decimal degrees, using the spatial reference system given in dwc:geodeticDatum) of the geographic center of a dcterms:Location. Positive values are east of the Greenwich Meridian, negative values are west of it. Legal values lie between -180 and 180, inclusive.
geodeticDatum	The ellipsoid, geodetic datum, or spatial reference system (SRS) upon which the geographic coordinates given in dwc:decimalLatitude and dwc:decimalLongitude are based.
coordinateUncertaintyInMeters	The horizontal distance (in meters) from the given dwc:decimalLatitude and dwc:decimalLongitude describing the smallest circle containing the whole of the dcterms:Location. Leave the value empty if the uncertainty is unknown, cannot be estimated, or is not applicable (because there are no coordinates). Zero is not a valid value for this term.
coordinatePrecision	A decimal representation of the precision of the coordinates given in the dwc:decimalLatitude and dwc:decimalLongitude.
georeferenceSources	A list (concatenated and separated) of maps, gazetteers, or other resources used to georeference the dcterms:Location, described specifically enough to allow anyone in the future to use the same resources.
dynamicProperties	A list of additional measurements, facts, characteristics, or assertions about the record. Meant to provide a mechanism for structured content.
georeferenceRemarks	Notes or comments about the spatial description determination, explaining assumptions made in addition or opposition to the those formalized in the method referred to in dwc:georeferenceProtocol.

### Data set 2.

#### Data set name

Occurrence Table

#### Data format

Darwin Core Archive

#### Character set

UTF-8

#### Download URL

http://ipt.gbif.pt/ipt/resource?r=arthropods_azores_darco

#### Data format version

1.7

#### Description

The dataset was published in the Global Biodiversity Information Facility platform, GBIF (Borges et al. 2025). The following data table includes all the records for which a taxonomic identification of the species was possible. The dataset submitted to GBIF is structured as an occurrence table that has been published as a Darwin Core Archive (DwCA), which is a standardised format for sharing biodiversity data as a set of one or more data tables. The core data file contains 1305 records (occurrenceID). This GBIF IPT (Integrated Publishing Toolkit, Version 2.5.6) archives the data and, thus, serves as the data repository. The data and resource metadata are available for download in the Portuguese GBIF Portal IPT (Borges et al. 2025).

**Data set 2.** 

Column label	Column description
eventID	An identifier for the set of information associated with a dwc:Event (something that occurs at a place and time). May be a global unique identifier or an identifier specific to the data set.
type	The nature or genre of the resource.
license	A legal document giving official permission to do something with the resource.
institutionID	An identifier for the institution having custody of the object(s) or information referred to in the record.
collectionID	An identifier for the collection or dataset from which the record was derived.
institutionCode	The name (or acronym) in use by the institution having custody of the object(s) or information referred to in the record.
collectionCode	The name, acronym, coden, or initialism identifying the collection or data set from which the record was derived.
datasetName	The name identifying the data set from which the record was derived.
basisOfRecord	The specific nature of the data record.
occurrenceID	An identifier for the dwc:Occurrence (as opposed to a particular digital record of the dwc:Occurrence). In the absence of a persistent global unique identifier, construct one from a combination of identifiers in the record that will most closely make the dwc:occurrenceID globally unique.
organismQuantity	A number or enumeration value for the quantity of dwc:Organisms.
organismQuantityType	A dwc:organismQuantityType must have a corresponding dwc:organismQuantity. This term has an equivalent in the dwciri: namespace that allows only an IRI as a value, whereas this term allows for any string literal value.
sex	The sex of the biological individual(s) represented in the dwc:Occurrence.
lifeStage	The age class or life stage of the dwc:Organism(s) at the time the dwc:Occurrence was recorded.
establishmentMeans	Statement about whether a dwc:Organism has been introduced to a given place and time through the direct or indirect activity of modern humans.
dynamicProperties	A list of additional measurements, facts, characteristics, or assertions about the record. Meant to provide a mechanism for structured content.
recordedBy	A list (concatenated and separated) of names of people, groups, or organizations responsible for recording the original dwc:Occurrence. The primary collector or observer, especially one who applies a personal identifier (dwc:recordNumber), should be listed first.
identfiedBy	A list (concatenated and separated) of names of people, groups, or organizations who assigned the dwc:Taxon to the subject.
dateIdentified	The date on which the subject was determined as representing the dwc:Taxon.
scientificName	The full scientific name, with authorship and date information if known. When forming part of a dwc:Identification, this should be the name in lowest level taxonomic rank that can be determined. This term should not contain identification qualifications, which should instead be supplied in the dwc:identificationQualifier term.
kingdom	The full scientific name of the kingdom in which the dwc:Taxon is classified.
phylum	The full scientific name of the phylum or division in which the dwc:Taxon is classified.
class	The full scientific name of the class in which the dwc:Taxon is classified.
order	The full scientific name of the order in which the dwc:Taxon is classified.
family	The full scientific name of the family in which the dwc:Taxon is classified.
genus	The full scientific name of the genus in which the dwc:Taxon is classified.
specificEpithet	The name of the first or species epithet of the dwc:scientificName.
infraspecificEpithet	The name of the lowest or terminal infraspecific epithet of the dwc:scientificName, excluding any rank designation.
taxonRank	The taxonomic rank of the most specific name in the dwc:scientificName.
scientificNameAuthorship	The authorship information for the dwc:scientificName formatted according to the conventions of the applicable dwc:nomenclaturalCode.
identificationRemarks	Comments or notes about the dwc:Identification.

## Additional information

In our comprehensive surveys across several arthropod groups, we documented a total of 92 taxa, with 75 having a species or subspecies level identification (see Table 2).

### Updates on the distribution of troglobiont endemic species

**Figure 2.**
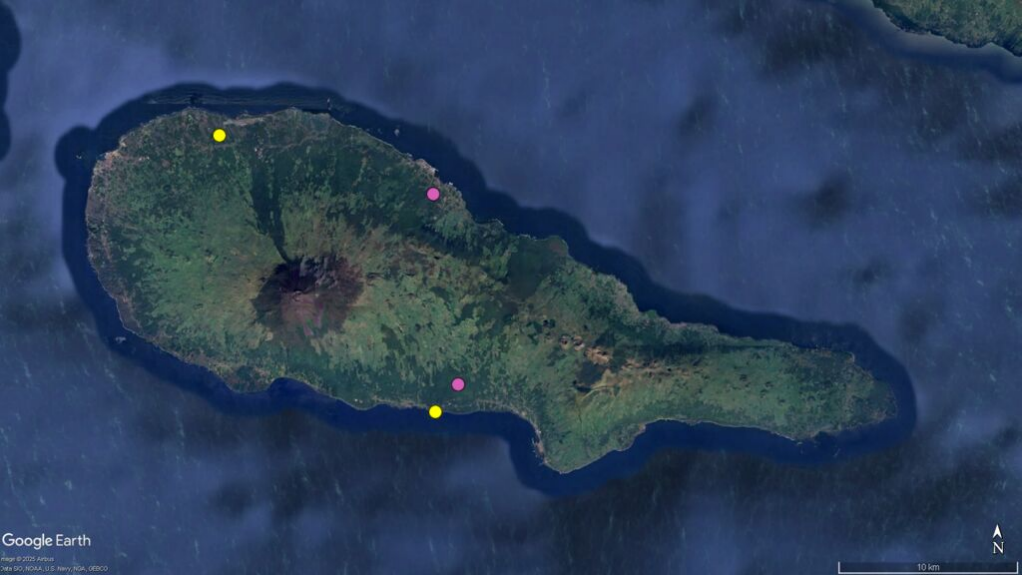
Map of Pico, with the distribution data of the endemic planthopper *Cixius azopicavus*. Legend: yellow circles - locations cited in taxonomic literature; pink circles - locations recorded for the first time. Image retrieved from Google Earth.

**Figure 3.**
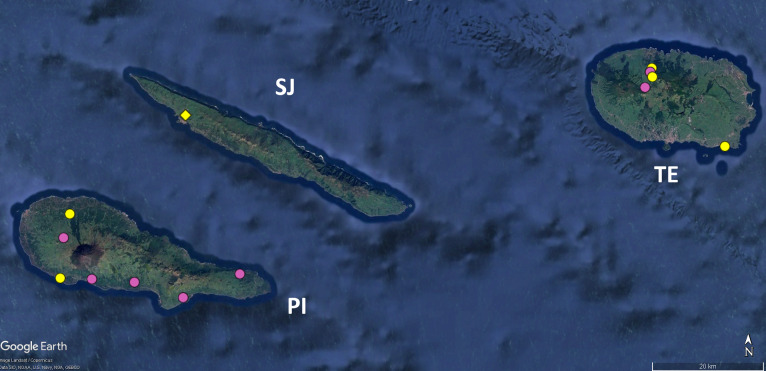
Map of the Central group of the Azores (except Graciosa and Faial), with the distribution data of the endemic pseudoscorpion species of the genus *Pseudoblothrus*. Legend: yellow circles - locations cited in taxonomic literature; pink circles - locations recorded for the first time in this study; circles - records of *P. vulcanus*; diamond - record of *P. oromii*. SJ - São Jorge, PI - Pico, TE - Terceira. Image retrieved from Google Earth.

The present work constitutes an attempt to tackle the "Wallacean shortfall" (Lomolino 2004) in the subterranean ecosystems of the Azores, with a special focus on the endemic cave-adapted species. These species are paramount to establish conservation priorities as was shown by Borges et al. (2012), which can be updated with the present data (Figs 1, 2, 3, 4). Particularly relevant was the increase in the known distribution of the centipide *Lithobius obscurus azoreae* (Fig. 1) and the pseudoscorpion *Pseudoblothrus vulcanus* (Fig. 3).

Other troglobiont species exist in the Azores, such as the spiders *Rugathodes pico* or the springtail *Pseudosinella ashmoleorum*, but we were unable to sample or to identify any specimen of these species. Additional points of interest rest, for instance, as referred above, in the strange distribution of both species of the pseudoscorpion genus *Pseudoblothrus*, which leads us to speculate on the result of a renewed taxonomic effort to study these taxa (Fig. 3).

Concerning the ground-beetles of the genus *Trechus*, three species are particularly geographically restricted, namely *T. oromii* from Faial and *T. jorgensis* and *T. isabellae*, both from São Jorge (Fig. 4a, b). In Pico and Terceira, we greatly increase the known distribution of *T. picoensis* (from three to nine sites) and doubled that of *T. terceiranus* (from five to ten sites), respectively (Fig. 4c, d). The phylogeography of these speciose endemics was studied by Amorim (Amorim 2005) and was the precursor for the majority of the sampling events reported in the present data.

### Conservation implications

Azorean subterranean adapted arthropods face significant conservation challenges due to their restricted distribution, small population sizes, habitat degradation, and climate change (Borges et al. 2012, Borges et al. 2019). These unique species, many of which are single island endemic, restricted to only a few caves and in some cases restricted to single caves, are highly vulnerable to environmental changes and human activities.

**Restricted Distribution and Population Size**

Many cave arthropods in the Azores have extremely limited ranges, often confined to one or two caves (Borges et al. 2012). This narrow distribution makes them particularly susceptible to habitat alterations, as a single disturbance event (e.g. a transformation of a forest to intensive pasture) can drastically reduce or even eliminate their populations. The isolation of these species further limits their ability to recolonize new habitats, increasing their extinction risk.

**Habitat Degradation and Land-Use Change**

One of the primary threats to Azorean cave arthropods is habitat degradation caused by human activities (Borges et al. 2019). Agricultural expansion, particularly intensive cattle production, has led to significant land-use changes. These activities contribute to soil erosion, alteration of nutrient cycles (e.g., nitrogen and phosphorus), and pollution, all of which can impact the delicate subterranean ecosystems where these arthropods thrive. Additionally, industrial *Cryptomeria japonica* plantations and small-holder farming continue to encroach upon native forests and underground habitats, further endangering these species.

**Tourism and Recreational Activities**

The increasing popularity of cave tourism in the Azores presents another major conservation challenge (Borges et al. 2019). Recreational activities, including cave exploration, can lead to physical disturbances that disrupt the fragile cave environments. The presence of tourists can alter the cave’s microclimate, introduce pollutants, exotic species and cause direct harm to arthropod populations and other cave biota. Without proper management, uncontrolled tourism can degrade the habitats that support these rare species.

**Climate Change and Severe Weather Events**

Climate change poses a long-term threat to Azorean cave arthropods by altering the stable environmental conditions above and within caves. Rising temperatures, changing humidity levels, and extreme weather events, such as prolonged droughts, could significantly impact cave ecosystems (Mammola et al. 2019). As these arthropods are highly specialized for stable, low-light and high humidity environments, even small climatic shifts could have profound effects on their survival and reproductive success negatively impacting the long-term survival of many unique species.

**Conservation Actions Needed**

To mitigate these threats, several conservation strategies must be implemented, following the example of Azorean terrestrial frorests (Borges et al. 2018):

1. **Regular Monitoring** – A systematic monitoring program should be established to track population trends and habitat conditions. By conducting assessments every five years, conservationists can identify early warning signs of population decline and habitat degradation.
2. **Protected Area Management** – Additional caves should be designated as protected areas, with restrictions on land-use activities surrounding these habitats. Fencing and controlled access to sensitive caves could help prevent disturbances from agriculture, livestock farming and tourism.
3. **Public Awareness and Education** – Raising awareness about the importance of subterranean ecosystems and in particular cave arthropods can help promote conservation efforts. Public engagement initiatives, such as macro photography exhibitions showcasing these unique species, could increase appreciation for their ecological role and the need for habitat protection.

Moreover, the effective conservation of Azorean cave arthropods requires targeted research strategies incorporating integrative taxonomy, population monitoring, habitat assessments, ecological interactions, genetics/genomics, climate change impacts and human disturbances. Integrative taxonomy, combining morphological, molecular, and ecological data, is essential for accurately identifying cryptic species (see e.g. Borges et al. 2017), assessing genetic diversity, and clarifying evolutionary relationships. Regular long-term surveys and non-invasive techniques like environmental DNA (eDNA) (Lunghi et al. 2022) analysis help track population trends, while habitat quality studies assess microclimate stability, pollution levels, and cave structural integrity. Understanding food web dynamics, predator-prey interactions, and invasive species presence ensures ecosystem balance (Mammola et al. 2024). Climate change studies should model microclimate shifts and species’ physiological tolerances to predict adaptation limits (Mammola et al. 2019). Research on human impacts must evaluate tourism effects, land-use changes, and conservation interventions using GIS mapping and remote sensing.

By integrating taxonomy, genetics/genomics, ecology, and environmental assessments, these research efforts will generate robust data to inform conservation planning, mitigate threats, and ensure the long-term survival of highly specialized and vulnerable Azorean cave arthropods (Borges et al. 2012).

Addressing the conservation challenges of Azorean cave arthropods requires a multifaceted approach that integrates habitat preservation, sustainable land management and public outreach. Without immediate action, these rare and highly specialized species face an uncertain future.

## Figures and Tables

**Figure 4a.**
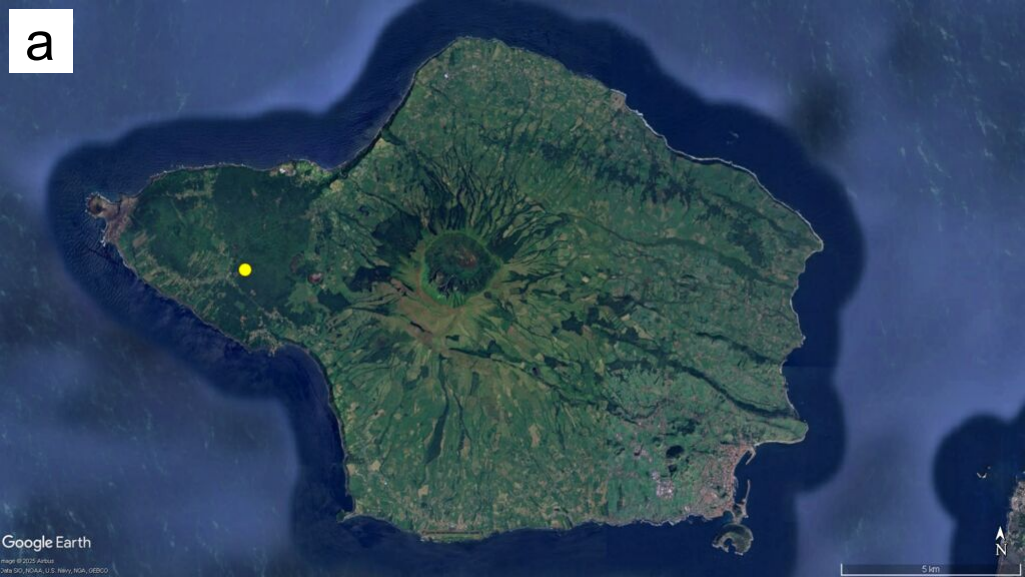
Map of Faial, with the single distribution record for *T. oromii* (yellow circle, taken from taxonomic literature).

**Figure 4b.**
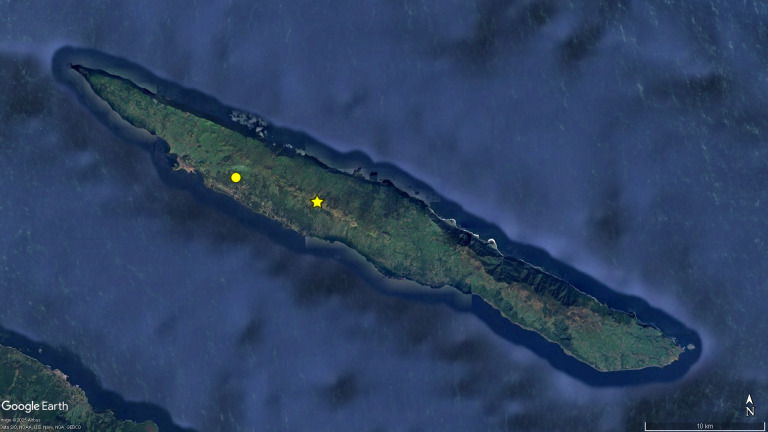
Map of São Jorge, with the single distribution records for *T. jorgensis* (yellow circle) and *T. isabelae* (yellow star) (both from taxonomic literature).

**Figure 4c.**
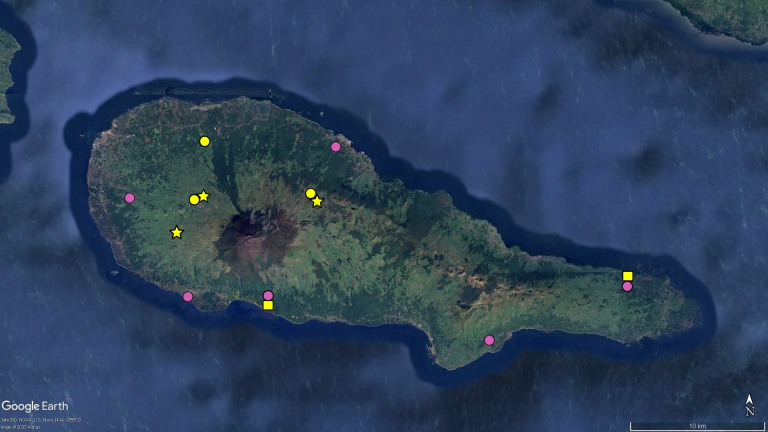
Map of Pico, with the distribution records for *T. picoensis* (circles), *T. montanheirorum* (stars) and *T. pereirai* (squares). Colors: yellow - records taken from taxonomic literature; pink - locations recorded for the first time in this study.

**Figure 4d.**
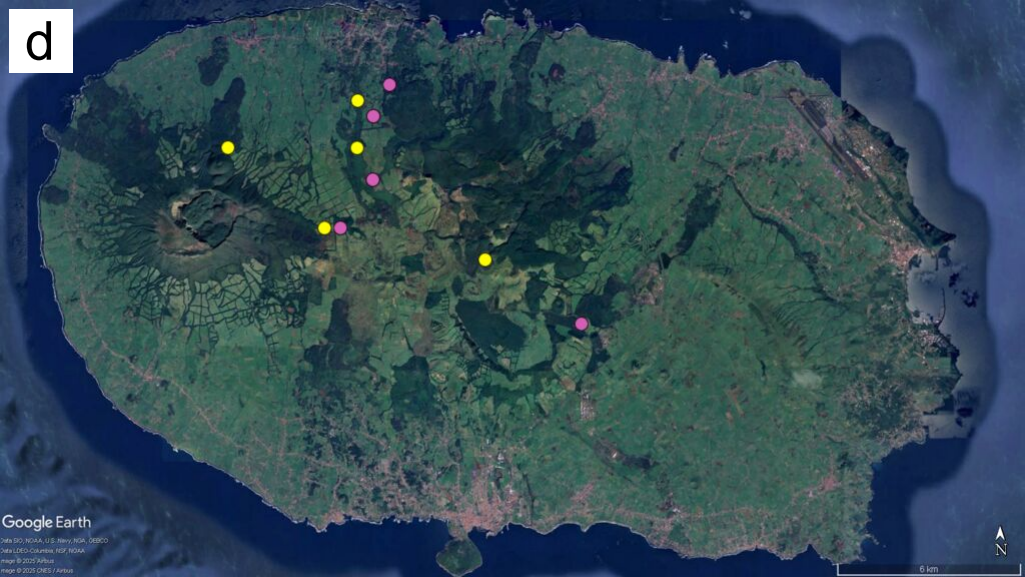
Map of Terceira, with the distribution records for *T. terceiranus* (circles). Colors: yellow - records taken from taxonomic literature; pink - locations recorded for the first time in this study.

**Table 1.** List of studied caves

island	locationRemarks	minimumElevationInMeters	decimalLatitude	decimalLongitude
Faial	Gruta das Anelares	150	38.53281	-28.7424
	Gruta do Cabeço do Canto	310	38.59824	-28.8115
	Gruta do Parque do Capelo	254	38.585607	-28.783055
Graciosa	Furna do Abel	168	39.030866	-27.984634
	Furna do Enxofre	98	39.024996	-27.972134
	Galeria do Forninho	95	39.02254	-27.99636
Pico	Furna da Baliza	10	38.430077	-28.457101
	Furna das Cabras II (terra)	180	38.429611	-28.391654
	Furna de Henrique Maciel	75	38.52806	-28.3337
	Furna do Frei Matias	690	38.49517	-28.4477
	Furna Nova	230	38.532665	-28.438024
	Gruta da Ribeira do Fundo	180	38.438205	-28.0908
	Gruta das Canárias	78	38.519149	-28.321732
	Gruta das Torres	285	38.49444	-28.505
	Gruta do Gabriel	150	38.401931	-28.205944
	Gruta do Mistério da Silveira I	301	38.42568	-28.30488
	Gruta do Soldão	10	38.41106	-28.3201
	Gruta dos Arcos	50	38.557445	-28.402493
	Gruta dos Cortiços	180	38.532189	-28.477721
	Gruta dos Montanheiros	770	38.49633	-28.3503
Santa Maria	Furna de Santana	10	37.003998	-25.159047
	Furna Velha	0	36.943229	-25.134684
São Jorge	Algar das Bocas do Fogo	385	38.67406	-28.16394
	Algar do Morro Pelado	1000	38.629269	-28.089117
	Gruta da Beira	258	38.69167	-28.20447
	Gruta da Recta da Cruz	480	38.651115	-28.014751
	Gruta das Raízes	494	38.658538	-28.0448
	Gruta do Leão	78	38.673357	-28.188644
	Gruta dos Encantados	404	38.73064	-28.24769
São Miguel	Gruta das Escadinhas	135	37.817949	-25.483239
	Gruta de Água de Pau	15	37.716026	-25.530134
	Gruta do Carvão	20	37.737863	-25.680924
	Gruta do Enforcado	235	37.813884	-25.692921
	Gruta do Pico da Cruz	260	37.785881	-25.624005
Terceira	Furna de Santa Maria	460	38.713502	-27.1817
	Gruta da Achada	317	38.729765	-27.151863
	Gruta da Branca Opala	255	38.78051	-27.2516
	Gruta da Madre de Deus	60	38.682982	-27.068984
	Gruta da Malha	505	38.751216	-27.254332
	Gruta das Agulhas	20	38.646006	-27.105721
	Gruta de Santo António	85	38.684782	-27.073585
	Gruta do Chocolate	250	38.780058	-27.2521
	Gruta do Coelho	540	38.739462	-27.271550
	Gruta do Natal	540	38.738566	-27.2692
	Gruta dos Balcões	395	38.763829	-27.2555
	Gruta dos Principiantes	335	38.770135	-27.2567

**Table 2.** List of identified species. Total refers to the total number of individuals identified. The Azorean Endemic species that are cave adapted species are in bold.

**Class**	**Order**	**Family**	**Scientific Name**	**Colonization**	**Total**
Arachnida	Araneae	Dysderidae	*Dysdera crocata* C. L. Koch, 1838	introduced	4
		Linyphiidae	*Canariphantes acoreensis* (Wunderlich, 1992)	endemic	1
			*Lessertia dentichelis* (Simon, 1884)	introduced	1
			*Oedothorax fuscus* (Blackwall, 1834)	introduced	1
			*Ostearius melanopygius* (O.Pickard-Cambridge, 1880)	introduced	1
			*Palliduphantes schmitzi* (Kulczynski, 1899)	native	1
			*Tenuiphantes tenuis* (Blackwall, 1852)	introduced	1
		Pholcidae	*Pholcus phalangioides* (Fuesslin, 1775)	introduced	5
		Salticidae	*Macaroeris cata* (Blackwall, 1867)	native	1
		Tetragnathidae	*Metellina merianae* (Scopoli, 1763)	introduced	2
		Theridiidae	*Cryptachaea blattea* (Urquhart, 1886)	introduced	1
			*Pholcomma gibbum* (Westring, 1851)	introduced	1
	Opiliones	Leiobunidae	*Leiobunum blackwalli* Meade, 1861	native	10
	Pseudoscorpiones	Chthoniidae	*Chthonius ischnocheles* (Hermann, 1804)	introduced	105
			*Ephippiochthonius tetrachelatus* (Preyssler, 1790)	introduced	101
		Syarinidae	***Pseudoblothrus vulcanus* Mahnert, 1990**	endemic	320
Chilopoda	Geophilomorpha	Geophilidae	*Geophilus truncorum* Bergsøe & Meinert, 1866	native	43
	Scutigeromorpha	Scutigeridae	*Scutigera coleoptrata* Linnaeus, 1758	introduced	4
	Lithobiomorpha	Lithobiidae	***Lithobius obscurus azoreae* Eason & Ashmole, 1992**	endemic	591
Diplopoda	Chordeumatida	Haplobainosomatidae	*Haplobainosoma lusitanum* Verhoeff, 1900	introduced	460
	Julida	Blaniulidae	*Blaniulus guttulatus* (Fabricius, 1798)	introduced	1325
			*Choneiulus palmatus* (Nemec, 1895)	introduced	1
			*Nopoiulus kochii* (Gervais, 1847)	introduced	1077
		Julidae	*Brachyiulus pusillus* (Leach, 1814)	introduced	1
			*Cylindroiulus latestriatus* (Curtis, 1845)	introduced	5
			*Cylindroiulus propinquus* (Porat, 1870)	introduced	9
			*Ommatoiulus moreleti* (Lucas, 1860)	introduced	2
	Polydesmida	Paradoxosomatidae	*Oxidus gracilis* (C.L.Koch, 1847)	introduced	6
		Polydesmidae	*Brachydesmus superus* Latzel, 1884	introduced	2
			*Polydesmus coriaceus* Porat, 1870	introduced	201
Insecta	Coleoptera	Carabidae	*Amara aenea* (DeGeer, 1774)	introduced	1
			*Laemostenus complanatus* (Dejean, 1828)	introduced	1
			*Ocys harpaloides* (Audinet-Serville, 1821)	native	1
			*Paranchus albipes* (Fabricius, 1796)	introduced	29
			*Pterostichus aterrimus aterrimus* (Herbst, 1784)	native	1
			***Thalassophilus azoricus* Oromí & Borges, 1991**	endemic	2
			***Trechus jorgensis* Oromí & Borges, 1991**	endemic	1
			***Trechus montanheirorum* Oromí & Borges, 1991**	endemic	25
			***Trechus oromii* Borges, Serrano & Amorim, 2004**	endemic	13
			***Trechus pereirai* Borges, Serrano & Amorim, 2004**	endemic	103
			***Trechus picoensis* Machado, 1988**	endemic	502
			***Trechus terceiranus* Machado, 1988**	endemic	159
		Cryptophagidae	*Cryptophagus cellaris* (Scopoli, 1763)	introduced	6
		Curculionidae	*Drouetius borgesi centralis* Machado, 2009	endemic	13
			*Otiorhynchus cribricollis* Gyllenhal, 1834	introduced	4
			*Pseudophloeophagus tenax borgesi* Stüben, 2022	endemic	1
		Elateridae	*Heteroderes azoricus* (Tarnier, 1860)	endemic	1
		Leiodidae	*Catops coracinus* Kellner, 1846	native	6
		Nitidulidae	*Epuraea biguttata* (Thunberg, 1784)	introduced	1
			*Stelidota geminata* (Say, 1825)	introduced	7
		Silvanidae	*Cryptamorpha desjardinsi* (Guérin-Méneville, 1844)	introduced	1
		Staphylinidae	*Aleochara verna* Say, 1833	indeterminate	20
			*Aloconota sulcifrons* (Stephens, 1832)	indeterminate	43
			*Anotylus nitidifrons* (Wollaston, 1871)	indeterminate	37
			*Atheta aeneicollis* (Sharp, 1869)	indeterminate	1
			*Carpelimus corticinus* (Gravenhorst, 1806)	indeterminate	3
			*Carpelimus zealandicus* (Sharp, 1900)	introduced	2
			*Creophilus maxillosus maxillosus* (Linnaeus, 1758)	indeterminate	3
			*Mocyta fungi* (Gravenhorst, 1806)	indeterminate	1
			*Ocypus olens* (O.F.Müller, 1764)	indeterminate	9
			*Proteinus atomarius* Erichson, 1840	indeterminate	1
			*Quedius curtipennis* Bernhauer, 1908	indeterminate	9
			*Quedius simplicifrons* Fairmaire, 1862	indeterminate	1
			*Sepedophilus lusitanicus* Hammond, 1973	indeterminate	1
	Dermaptera	Anisolabididae	*Euborellia annulipes* (Lucas, 1847)	introduced	4
	Hemiptera	Aphididae	*Rhopalosiphoninus latysiphon* (Davidson, 1912)	introduced	8
		Cicadellidae	*Eupteryx azorica* Ribaut, 1941	endemic	1
		Cixiidae	***Cixius azopicavus* Hoch, 1991**	endemic	6
			*Cixius azopifajo azopifajo* Remane & Asche, 1979	endemic	12
			*Cixius azoterceirae* Remane & Asche, 1979	endemic	1
		Lygaeidae	*Kleidocerys ericae* (Horvath, 1909)	native	1
		Rhyparochromidae	*Scolopostethus thomsoni* Reuter, 1874	native	1
	Hymenoptera	Formicidae	*Lasius grandis* Forel, 1909	native	14
			*Linepithema humile* (Mayr, 1868)	introduced	3
			*Monomorium carbonarium* (Smith, 1858)	native	1
